# Highly Stable and Efficient Performance of Binder-Free Symmetric Supercapacitor Fabricated with Electroactive Polymer Synthesized via Interfacial Polymerization

**DOI:** 10.3390/ma12101626

**Published:** 2019-05-17

**Authors:** Muhammad Fahim, Anwar ul Haq Ali Shah, Salma Bilal

**Affiliations:** 1 National Centre of Excellence in Physical Chemistry, University of Peshawar, Peshawar 25120, Pakistan; fahim_kn@yahoo.com; 2 Institute of Chemical Sciences, University of Peshawar, Peshawar 25120, Pakistan; anwarulhaqalishah@uop.edu.pk; 3 TU Braunschweig Institute of Energy and Process Systems Engineering, Franz-Liszt-Straße 35, 38106 Braunschweig, Germany

**Keywords:** polyaniline, interfacial polymerization, gasoline, symmetric supercapacitor, charge storage/release mechanism

## Abstract

The use of electroactive polyaniline (PANI) as an electrode material for a symmetric supercapacitor has been reported. The material was synthesized via interfacial polymerization, using ammonium per sulfate, dodecylbenzene sulfonic acid (DBSA), and gasoline, respectively, in the oxidant, dopant, and novel organic phase, and was subsequently employed as an electrode material to design a binder-free symmetric capacitor. As properties of PANI rely on the method of synthesis as well as reaction parameters, the present combination of reactants, at pre-optimized conditions, in the interfacial polymerization, led to the formation of PANI exhibiting a high specific capacitance (712 Fg^−1^ at 0.5 Ag^−1^), a good rate capability (86% capacitance retention at 10 Ag^−1^), a very low solution resistance (Rs = 0.61 Ω), and a potential drop (IR = 0.01917 V). The device exhibited a high energy density of 28 Whkg^−1^, at a power density of 0.28 kWkg^−1^, and retained as high as 15.1 Whkg^−1^, at a high power density of 4.5 kWkg^−1^. Moreover, it showed an excellent cycling stability and retained 98.5% of coulombic efficiency after 5000 charge discharge cycles, without showing any signs of degradation of polymer.

## 1. Introduction

Electrochemical capacitors, due to their unique properties, including environmental friendliness, high specific power, cyclability, and fast charging/discharging rate, are considered to be the promising power source for a vast variety of applications, such as electric and hybrid electric vehicles and power backup systems for emergency use, etc. [1,2,3]. Electrode material in such energy storage system, determines its efficiency, delivery rate, and capability. Based on the electrode materials, the supercapacitors are grouped into Electrical Double Layer Capacitors (EDLCs) and pseudocapacitor [4,5,6,7]. Both, pseudocapacitors and EDLCs require the electrode materials with good electrical and morphological properties, for a high performance energy storage application.

Conducting polymers [8,9,10], particularly polyaniline (PANI), due to its easy synthesis, eco-friendliness, frugal, and reversible redox acid/base properties, has been extensively tested as highly active capacitive electrode materials. Since it exhibits variable potential dependent oxidation states, transition to different states leads to a very high value of theoretical capacitance. Nano dimensional PANI-based electrode materials have been reported to exhibit a high capacitance, which strongly rely on their morphologies [2,11,12].

However, PANI in the context of energy storage applications still suffer from four major problems—(1) low specific capacitance, (2) low charge storage stability, i.e., at a particular current density upon multiple charge–discharge cycles its capacitance dies out rapidly, (3) fading of capacitance with current density growth, and (4) need of polymeric binder for electrode fabrication.

For instance, Radhakrishnan et al. reported the capacitive properties of PANI–DBSA synthesized through precipitation oxidation and fabricated a super capacitor with PANI–DBSA–Fe_3_O_4_ nanocomposites [13]. The device showed a low capacitance of 180–135 F/g at 1–5 mA/cm^2^. The charge–discharge tests carried out for 1000 cycles, at a current density of 5 mA/cm^2^, showed that the capacitance value falls to 84 F/g in the last charge–discharge cycle. The decrease, from the first to 1000th, was about 35% and it was suggested that there is a degradation of electrolytes and PANI. 

It is an established fact that properties of PANI exceptionally depend upon the method of synthesis and different factors, such as type of solvent system, dopant, and oxidant, etc. [14,15,16]. Even PANI, doped with the same dopant but, synthesized through a different route will exhibit a different solubility of morphological and electrochemical properties, and vice versa [16,17].

Amongst many routes, polymerization of aniline through interfacial polymerization has been reported to be a good choice to obtain PANI with controlled properties, and without the need of any post treatment [18]. The method is considered to provide an ideal confined environment for the growth of polymer exhibiting different morphological features, and has been successfully employed by different groups to obtain polymeric materials in the form of nanoneedles, nanofibers, or nanorods, which have successfully been utilized for their supercapacitive properties [19,20,21,22,23,24].

In the past decade, dodecylbenzene sulfonic acid (DBSA) has emerged as a very promising candidate to be used as dopant or emulsifier for the synthesis of PANI, and is still a good choice for researchers [14,16,17,25,26,27]. Most often, the PANI material shows valuable properties when DBSA is added to the reaction bath, regardless of the method of synthesis [13,14,15,16,17,23,28]. 

Herein, we report the synthesis of PANI through an interfacial polymerization route in which DBSA was used as a dopant, ammonium per sulfate (APS) was used as an oxidant, while gasoline was employed as a novel organic phase. This combination turned out to be quite effective and the resulting PANI showed excellent capacitive properties, while the device fabricated with it was highly stable. Moreover, electrode fabrication did not need any binder, making the process simpler.

Although, a large number of papers is available on the use of different PANI based materials in supercapacitors, a comparison of the capacitive properties of PANI synthesized via different routes has been carried out for the current work, and is presented in Table 1 [29,30,31,32,33,34]. To the best of our knowledge, there is no report on the fabrication of a symmetric supercapacitor with PANI synthesized via interfacial polymerization, utilizing gasoline as an organic solvent that is doped with DBSA. In order to better understand this process and to confirm the respective roles of DBSA and gasoline, an extensive comparative study is underway to replace the dopant DBSA, with other surfactant molecules and inorganic acids (another common class of dopants), and organic solvent gasoline by other commonly used organic solvents, such as chloroform, toluene, acetone, etc., and will be presented in near future.

## 2. Experimental

### 2.1. Materials

Ammonium persulfate (APS), acetone, sulfuric acid, H_2_SO_4_ (98%), toluene, 2-propanol (Scharlu), chloroform, dodecyl benzenesulphonic acid, DBSA, (sigma), and gasoline (Shell) were used as received. Aniline (Sigma) was double-distilled and stored under nitrogen. All chemicals were of the analytical grade. All solutions were prepared using deionized water.

### 2.2. Methods

#### 2.2.1. Synthesis of PANI–DBSA

Polyaniline doped with DBSA (PANI–DBSA) was synthesized through interfacial polymerization, e.g., an organic solution (commercially available gasoline) containing 0.1 M aniline and 0.35 M APS in 15 mL of water, with DBSA (0.2 M) acting as the organic and aqueous phases, respectively. The monomers and oxidant ratio were kept at 1:3.5. The aqueous phase was stirred for 10 min, to form a uniform dispersion, then, the organic phase was added carefully, along the wall, to a beaker containing an aqueous phase. The polymerization was carried out without any disturbance, for 24 h. After ∼24 h, the product was repeatedly washed with acetone and water, until the washings were colorless. The products were dried at room temperature for 24 h.

#### 2.2.2. Structural Characterization

FTIR spectra were recorded with IRAffinity-1S Fourier Transform Infrared spectrophotometer (Shimadzu, Tokyo, Japan), scanning over the wavenumber range of 400–4000 cm^−1^, with 2 cm^−1^ resolution. Surface imaging and elemental mapping of the synthesized samples was performed through SEM and an SEM–Energy Dispersive X-ray (EDX) analysis (Helios G4 CX Dual Beam microscope equipped with Octane Elite, EFI Berlin Germany). Atomic force microscopic imaging was done through NanoWizard® 3 Bio Atomic Force Microscope (JPK/Bruker, Berlin Germany). The test samples for electrical conductivity was prepared in a pellet form (diameter—10 mm, thickness—0.5 mm), at a pressure of 80 MPa. Four probe methods was used to measure the electrical conductivity of samples by a Jandel RM3000 four-probe resistivity/square resistance tester (Bridge Technology Co. Ltd., Chandler Heights Arizona, USA)

#### 2.2.3. Electrochemical Characterization

Electrochemical performance was evaluated using a standard three electrode setup, using 1 M H_2_SO_4_ as electrolyte. Concentrated PANI–DBSA dispersed in a mixture of 2-propanol and toluene (1:3), was sprayed on a gold sheet, and dried, to form a working electrode. The electrode preparation time was 2 min. Ag/AgCl and gold sheet were used as reference and counter electrodes, respectively. The mass loading of PANI–DBSA on the working electrode was 0.35 mg. Cyclic Voltammograms (CVs) were registered at different scan rates, ranging from 5–200 mVs^−1^, in a potential range of 0.0–0.75 V. Electrochemical Impedance Spectroscopic (EIS) data were collected at the open circuit potential, with an AC perturbation of 5 mV, for swept frequencies of 0.1 Hz–100 kHz. Galvanostatic Charge Discharge (GCD) measurements were performed at various current densities (0.5–10 Ag^−1^). Cycling stability was tested by charge–discharge, at a constant current density of 10 Ag^−1^. All CV, EIS, GCD experiments were performed at room temperature (22 ± 3 °C), by means of ZRA potentiostat/galvanostat Reference 3000 (Gamry, Warminster, PA, USA), using PHE-200, EIS-3000, and DC-105. The Specific capacitance, Cs, (Fg^−1^) from the three electrode setup was calculated from CV and GCD, using Equations (1) and (2), respectively.

(1) $$\mathrm{Cs}=\frac{1}{\mathrm{vm}\left(\Delta V\right)}\int_{\mathrm{Va}}^{\mathrm{Vc}}\mathrm{iVdV}$$

(2) $$\mathrm{Cs}=\;\frac{I}{m\;(\Delta V/\Delta t)}$$

where ΔV is the applied potential window (Va to Vc), v is the scan rate, m is mass of the active material, and I (A) and ∆t (s) represent the current response and time during the discharging process. The relaxation time (τo) was calculated from the bode plot of EIS, using Equation (3)

(3) $$\tau o=\;\frac{1}{f_{o}}$$

where f_o_ is the frequency at a phase angle of −45° [35]. Capacitance (C) for the EIS curve was calculated using Equation (4)

(4) $$C=\;-1/2\pi {\mathrm{fZ}}^{\prime \prime }$$

where f is frequency and Z″ is the corresponding imaginary resistance [36].

To demonstrate a practical application, PANI–DBSA was assembled into two symmetric electrode setup, to form a supercapacitor device, represented by P–DBSA ║ P–DBSA. In brief, the device consisting of the two identical pieces of PANI–DBSA was coated on the current collector (gold sheet of 1 × 1 cm^2^) with a similar charge capacity, as discussed above. The filter paper of ∼25 μm thickness and wetted by 1 M H_2_SO_4_ was chosen as a separator and was sandwiched between the two electrodes. The entire device configuration was then enclosed in an epoxy coating, to mitigate the electrolyte evaporation processes. The specific capacitance, Cs, (Fg^−1^) derived from the GCD curve, was calculated from the discharge time, after the potential drop (V_IR_) (V_IR_ was mainly attributed to the intrinsic resistance of the electrode material and the resistance of the electrolyte), based on Equation (5) [37]

(5) $$\mathrm{Cs}=\;\frac{4\;I\;\Delta t}{m\;\left(\Delta V-V_{\mathrm{IR}}\right)}$$

Equivalent series resistance (R_ESR_) was calculated using the voltage drop at the beginning of the discharge curve V_drop_ (V), at constant current I (A), using Equation (6)

(6) RESR=Vdrop2I

The energy density (ED) and power density (PD) were calculated using Equations (7) and (8), respectively.

(7) $$\mathrm{Cs}=\frac{1}{\mathrm{vm}\left(\Delta V\right)}\int_{\mathrm{Va}}^{\mathrm{Vc}}\mathrm{iVdV}$$

(8) $$\mathrm{Cs}=\frac{1}{\mathrm{vm}\left(\Delta V\right)}\int_{\mathrm{Va}}^{\mathrm{Vc}}\mathrm{iVdV}$$

where Cs is the specific capacitance from GCD, ΔV is the applied potential window, V_IR_ is the potential drop, M is the total mass of the active material on two electrode, and R_ESR_ is the equivalent series resistance.

## 3. Results

### 3.1. Structural Characterization

**Figure 1:**
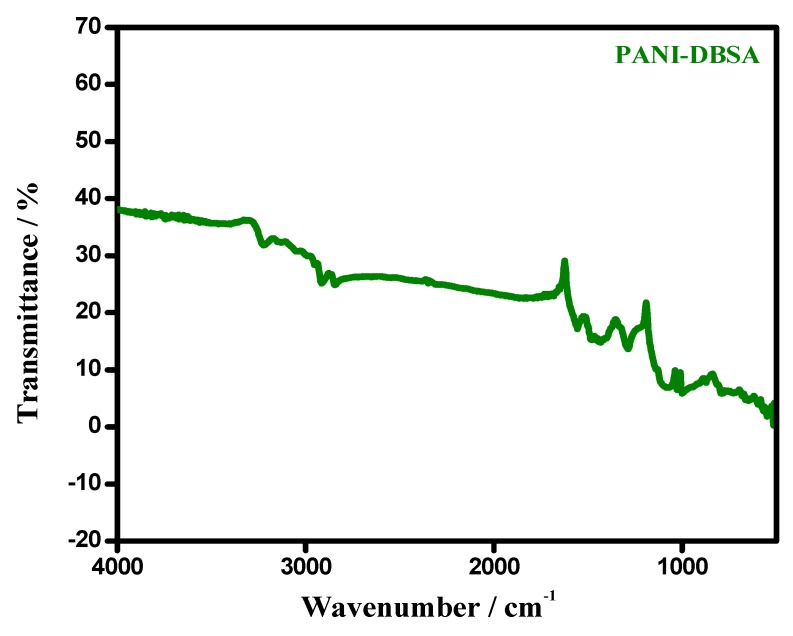
FTIR spectrum of the synthesized polyaniline (PANI).

The FTIR spectrum recorded for PANI–DBSA is portrayed in Figure 1. It shows the characteristic bands corresponding to the stretch of quinoid and benzenoid ring, at 1564–1468 cm^−1^, endorsing the successful synthesis of PANI. The intensity ratio (I_Q_/I_B_) of quinoid and benzenoid absorption peaks, specify the oxidation state of PANI, i.e., demonstrating the comparative content of quinoid diamine and benzene ring structures. A higher intensity ratio of 0.51 reveals the greater conductivity, and reflects the conductive state (emeraldine salt form) of PANI [38]. Furthermore, weak characteristic features corresponding to the polaron (1240 cm^−1^) and para-coupling structure (838 and 513 cm^−1^), were also present. A summary of all the other bands along with their assignments, is provided in Table S1.

**Figure 2:**
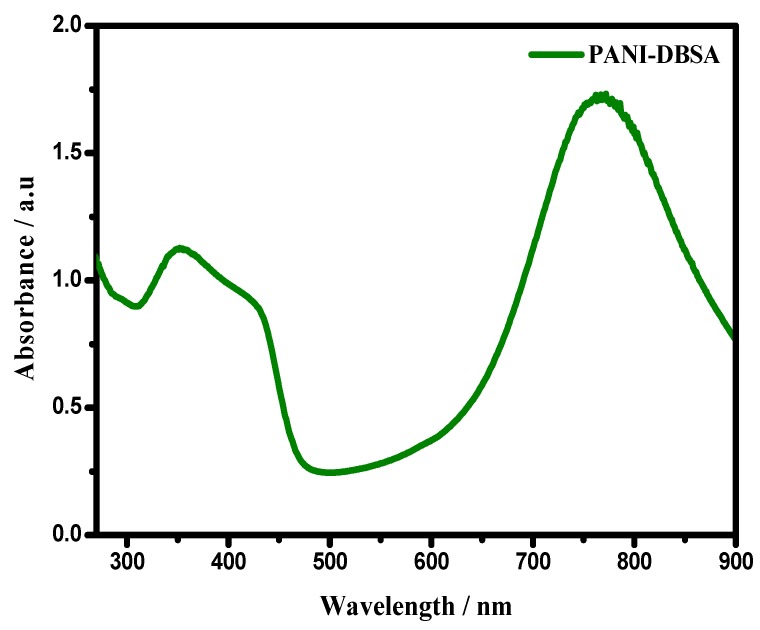
UV/Vis spectrum of PANI–dodecylbenzene sulfonic acid (DBSA).

Figure 2 shows the UV–Vis absorption spectrum of the synthesized PANI. The spectrum endorses the successful synthesis of PANI, by reflecting two characteristic absorption bands at 316–360 and 420–490, ascribed to π–π* and the polaron-π* transitions, respectively. The absorption around 780–800 nm was assigned to the presence of polaron resulting from the doping process. This indicated the conducting emeraldine salt form of PANI [39,40]. The conductivity of PANI–DBSA was found to be 0.0671 Ω^−1^ cm^−1^.

**Figure 3:**
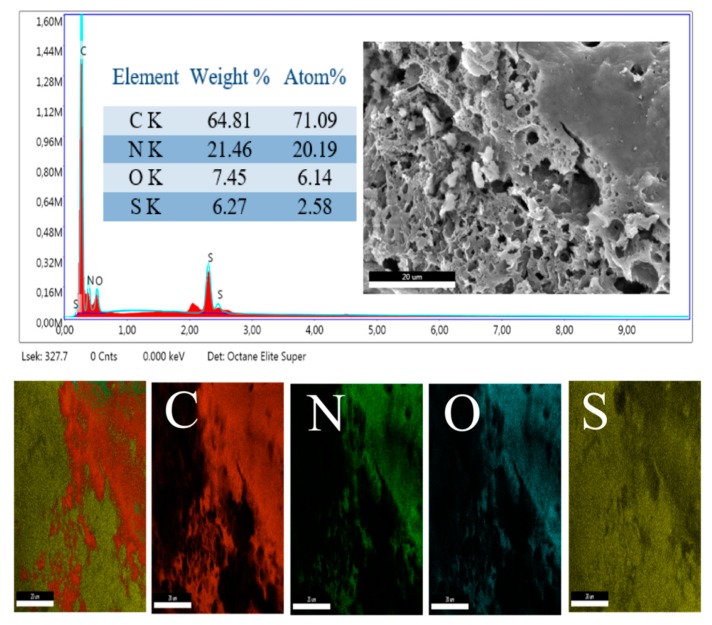
Elemental composition and SEM–EDX spectrum of the PANI–DBSA sample, along with the mapping of the elements on the polymer surface.

The structural composition of the PANI skeleton and successful incorporation of the doping species on the backbone, was further confirmed by an elemental analysis. Figure 3 depicts the SEM–EDX spectrum and the corresponding elemental composition of mapping of the different elements, on the PANI surface. The spectra show the high contents of carbon, which is the cardinal element of the PANI backbone and hydrocarbon affixes, with the main chain, in the case of the organic dopants. Furthermore, the presence of Sulfur (S) and Oxygen (O) in the spectra’s, evidenced the successful attachment of the doping species onto the polymer backbone. It could be observed that the S element showed peaks at three different positions—2.308, 2.47, and 0.0193 keV. These peaks could be assigned to K*α*_1_, K*edge*, and L*_I_edge*, respectively [41]. For a particular absorbing element, a sharp rise was observed when the energy of the incident photon was equal to the binding energy of an electron shell either K, L, or M, etc., in the absorber. This was the least energy for the creation of a vacancy in the particular shell. This energy was referred to as the ‘critical excitation’ or ‘edge’. When the ‘initial’ vacancy in an inner shell, created by electron or X-ray, was filled by electron transfer from another shell, it left a ‘final’ vacancy in that shell; and the characteristic X-ray lines were generated. The energy of the line was equal to the difference in binding energies of the shells, where the ‘initial’ and ‘final’ vacancies were generated. The X-ray spectra from an element could include lines from the K, L, M, N, and O series, corresponding to the excitation of the K, L, M, N, or O levels, based on the atomic number. It has been reported that the incorporation of the S elements in the mesoporous carbon, led to the enhancement of its energy conversion and storage capability [42,43].

**Figure 4:**
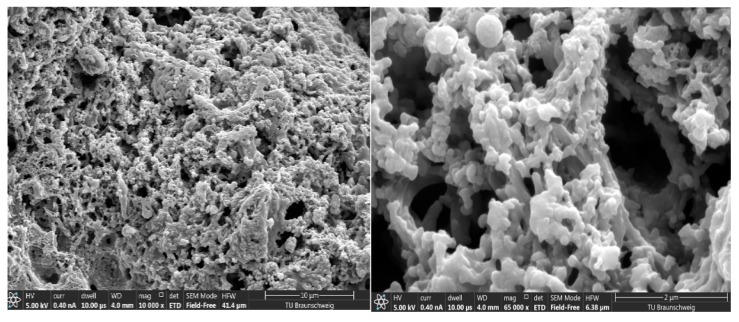
SEM images of PANI–DBSA at a magnification of 10,000 (left) and 65,000 (right).

Figure 4 shows the SEM analysis of PANI–DBSA. It showed coral reef-like morphology and seemed to be highly porous. The advantage of polymerizing aniline, via interfacial polymerization, lies in the fact that the secondary growth and aggregation of the polymer chain, due to ortho-coupling reactions, are avoided by favoring 1–D morphology. The porous nature of the polymer could further be assisted by Atomic Force Microscopic Analysis (Figure S1), where the appearance of the dark regions, clearly indicate the presence of pores or deep areas into the polymer surface.

### 3.2. Cyclic Voltammetry

**Figure 5:**
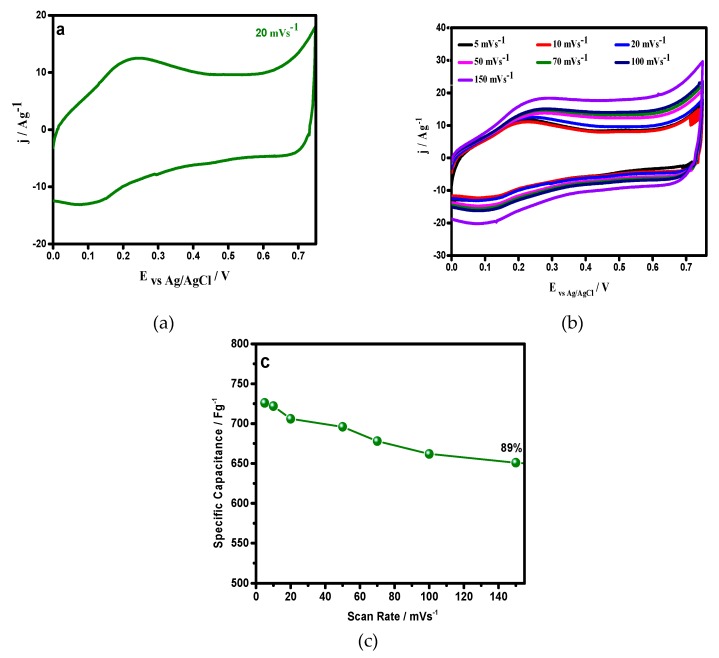
Cyclic Voltammograms (CVs) of the recorded PANI–DBSA (**a**) at 20 mV/s. (**b**) In a scan range of 5–150 mVs^−1^, as indicated, and (**c**) capacitance retention of PANI–DBSA as a function of the scan rate.

Before the application of the synthesized material in symmetric supercapacitors, its electrochemical properties were evaluated in a three electrode setup, using 1 M H_2_SO_4_ as electrolytes [Figure 5]. Cyclic voltammograms (@ 20 mVs^−1^ (Figure 5a) depicts that a first anodic signal appeared at 0.25 V and the start of a second signal was observed at 0.75 V. Both signals were typical of the electrodes with PANI conduction. The corresponding cathodic signals were clearly observed in the profile at 0.1 V and 0.69 V. However, beyond 0.25 V, the CV shape became rectangular, indicating a capacitive nature, resulting from the synergistic effect of, both, pseudocapacitance and a double layer capacitance [44,45]. The material exhibited a high specific capacitance, large integrated area outstanding rate capability, with a scan rate growth from 5 to 150 mVs^−1^ (Figure 5b,c). It seemed that it favored a fast ionic transportation rate, with a remarkable capacitance at a high current density [46].

### 3.3. Galvanostatic Charge Discharge (GCD)

**Figure 6:**
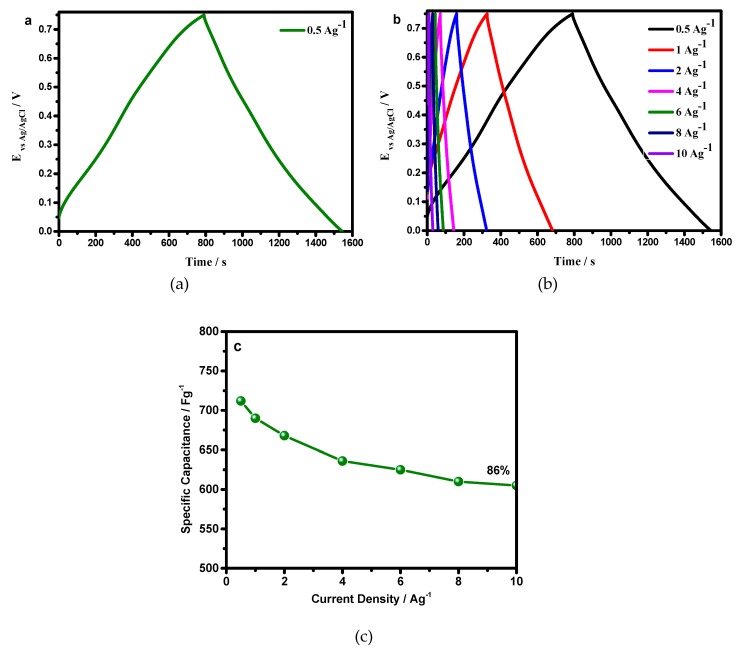
Galvanostatic Charge Discharge (GCD) curves of PANI–DBSA, (**a**) at 0.5 Ag^−1^, (**b**) at a different current density, ranging from 0.5–10 Ag^−1^, and (**c**) rate capability with a current density growth from 0.5–10 Ag^−1^.

The charge storage phenomenon for the present system was systematically studied. The GCD curve exhibited a symmetric, triangle shape charge discharge profile, indicating an excellent reversibility, and a capacitive behavior of the material (Figure 6a). The material had a lesser potential drop <0.01917 V at 0.5 Ag^−1^, which indicated an availability of more faradaic centers to the electrolyte, hence, it minimized useful voltage losses. The IR drop data analogized, quite well, to morphological features.

Figure 6b shows the specific capacitance behavior with increased current density. The specific capacitance remained as high as 605 Fg^−1^, at a current density of 10 Ag^−1^, corresponding to a capacitance retention of 85%, with a growth of current density from 0.5 to 10 Ag^−1^, suggesting an excellent rate capability (Figure 6c). These properties suggested that PANI–DBSA afforded a smooth flow of electrolytic ions, resulting in a low IR drop, even at a high current density of 10 Ag^−1^.

### 3.4. Electrochemical Impedance Spectroscopy (EIS)

EIS is the most well-grounded, powerful diagnostic tool to extricate electrochemical characteristics encompassing solution resistance (Rs), charge transfer resistance (Rct), double layer capacitance (cdl), and processes occurring at the electrode/electrolyte interface, i.e., charge transport and diffusion impedance processes. The fast process occurring at the PANI/electrolyte interface was sensible for the impedance at high frequency regions, whereas the slow process in the PANI film, corresponded to a low frequency domain. It was notable that the PANI impedance responses were significantly influenced by its properties. Depending on the model applied to the data, the EIS parameters were susceptible to different interpretations.

**Figure 7:**
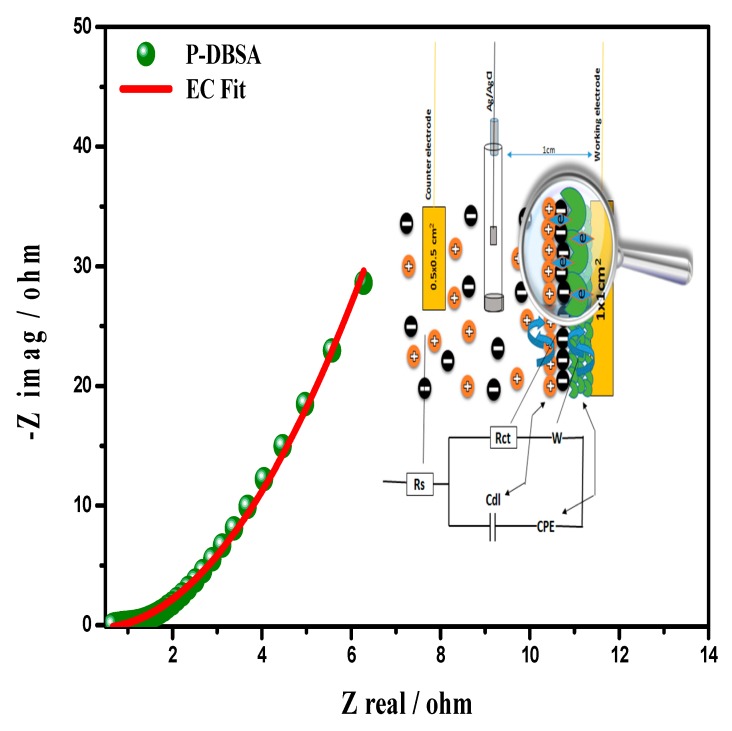
Nyquist plot of the PANI–DBSA and the best-fitted equivalent circuit with mechanisms for elemental assignment.

Figure 7 show the Equivalent Circuit (EC), best fitted on the experimental data of the Nyquist plot (imaginary impedance—z″, vs. real impedance—z′), for the synthesized PANI–DBSA. The left lower portion represents the higher frequency response, whereas the line parallel to the imaginary impedance, surged vertically, endorsing a negligible Warburg resistance, a high capacitive character, and a fast double layer formation. Equivalent series resistance (ESR) (caused by the Rs and the internal resistance of the electrode) could be obtained from the Nyquist plot, where the semicircle on the real axis, cuts at the high frequency end. The system showed the lowest ESR value, showing an ideal capacitive behavior, ascribed to the low diffusion resistance offered by the electrode material. Moreover, low ESR value is very important to decrease the IR drop of supercapacitors charged and discharged at high current densities. The diameter of the semicircle is the Rct, remarking the interfacial processes of the counter ions through the electrode/electrolyte interface. Indeed no semicircle was observed at the high frequencies endorsing the fast electronic diffusion, with a high conductivity.

**Figure 8:**
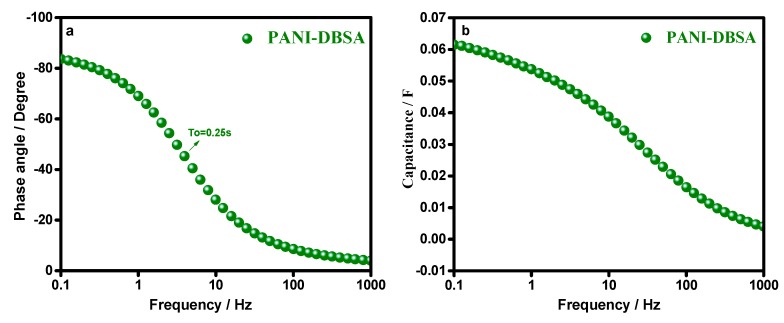
(**a**) Bode Plot with a relaxation time constant (τ_o_) and (**b**) capacitance vs. frequency response, of the synthesized PANI–DBSA.

In Figure 8a,b, the Bode plot and capacitance vs. the frequency plot, respectively, suggested that the synthesized material was highly capacitive, at a phase angle of −83°, resembling the ideal capacitive behavior. A high capacitance was retained in a broad frequency region, more than 50% of capacitance was achieved upto 25 Hz.

### 3.5. Application of the Synthesized PANI–DBSA in a Symmetric Supercapacitor

Exemplifying the scaling up potential toward a practical application, a symmetrical supercapacitor device based on the use of synthesized PANI–DBSA as an electrode material, designated as P–DBSA ║ P–DBSA, was fabricated. 

**Figure 9:**
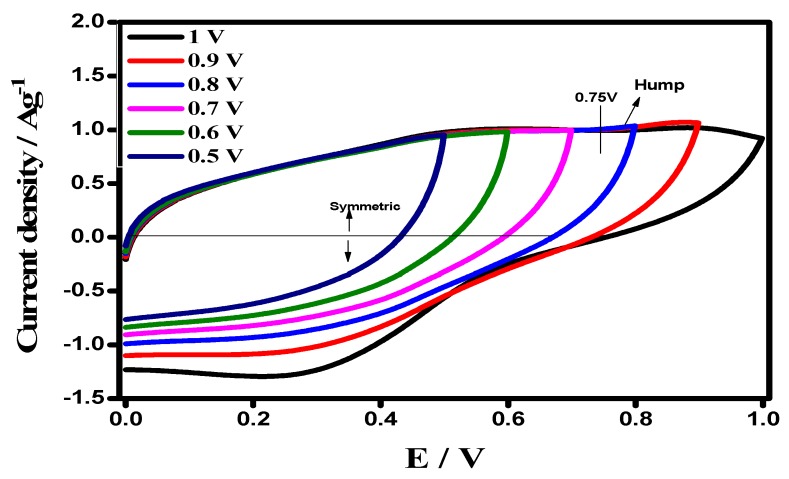
CV of the symmetric device in the upper potential range 0.5–1.0 V, keeping a lower potential at 0.0 V.

The CV of device in different potential ranges (0.0 V to 0.5–1 V), at a scan rate of 50 mV s^−1^, are depicted in Figure 9. The voltammograms exhibit a rectangular shape from 0.0–0.75 V, which is a typical characteristic of an ideal capacitive nature, resulting from the synergistic effect of, both, a double layer capacitance and pseudocapacitance. However, above the potential cut-off of 0.75 V, a slight hump and distortion in the CV curve appeared at around 0.8 V, endorsing some irreversible reaction. 

**Figure 10:**
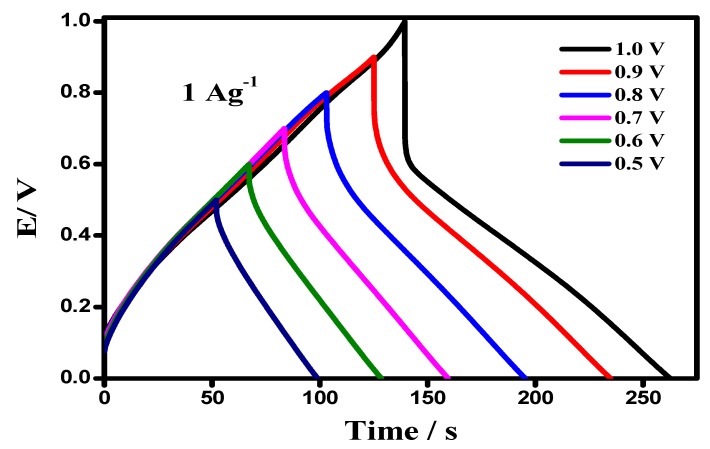
GCD of a device with varying potential, from 0.0 V to 1.0 V, at an interval of 0.1 V.

**Figure 11:**
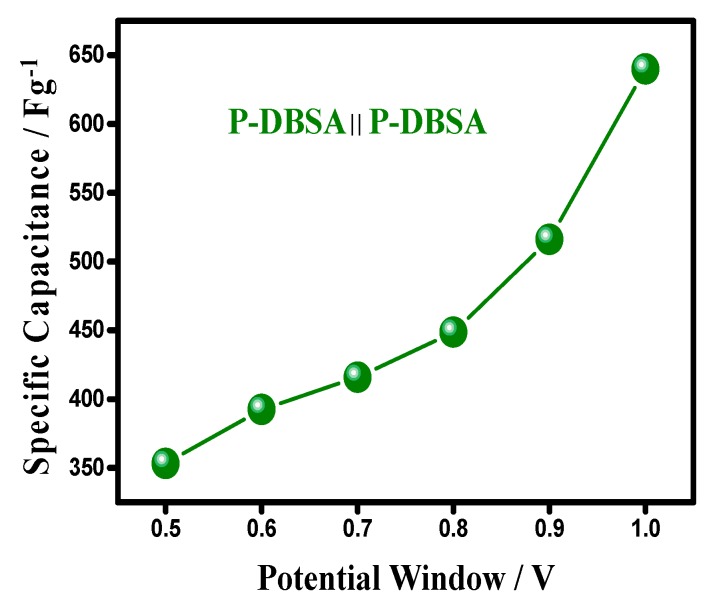
Increase in the upper potential resulting in the enhancement of a specific capacitance.

The GCD of a device with an upper potential variation from 0.5–1.0 V, keeping a lower potential at 0.0 V at a current density of 4 A g^−1^, is shown in Figure 10. The curve showed a linear discharge shape, indicating a highly capacitive behavior and a well-balanced charge storage. However, above the potential cut of 0.0–0.75 V, an increase in potential drop was observed, resulting in useful voltage losses. The symmetrical device showed a Csp value of 640 Fg^−1^, keeping the cut-off potential window at a maximum 1 V, whereas, the Csp at an optimized potential window was 415 Fg^−1^ at 50 mV s^−1^, shown in Figure 11. The attribution in capacitance to a higher potential had resulted due to an irreversible reaction and an asymmetric feature, favoring a more characteristic feature of a battery-type material, than a supercapacitor.

**Figure 12:**
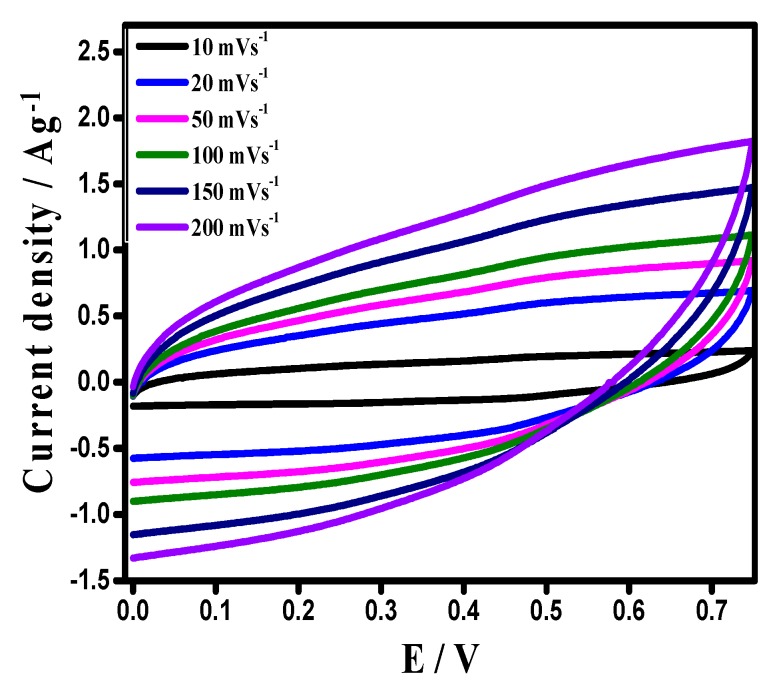
CVs of the device at different scan rates on best optimized potential window.

Thus, the optimized potential window for a device can be from 0.0–0.75 V. The shape of the CV curve was a mirror symmetric at a cut-off potential window of 0.75 V, at a high scan rate, indicating an excellent reversibility and capability of the assembled device (Figure 12).

However, the discharge curve was mirror symmetric with a negligible IR drop at an optimized potential range, which is a typical characteristic of a supercapacitor, as reported earlier. The linearity of the charging/discharging curve was retained, even at a high current density varyinh from 1–10 A g^−1^, indicating an excellent reversibility of the device. The Csp was 412 Fg^−1^ @ 1 Ag^−1^, which remained as high as 215 Fg^−1^ @ 10 Ag^−1^, corresponding to an excellent rate capability of the device to deliver a high power. The high Csp value was envisaged as favoring the fastest electrolyte ion transportation, by the PANI–DBSA, probably utilizing, both, the outer and the inner surfaces for the intercalation of ion, resulting in high a Csp and rate capability.

**Figure 13:**
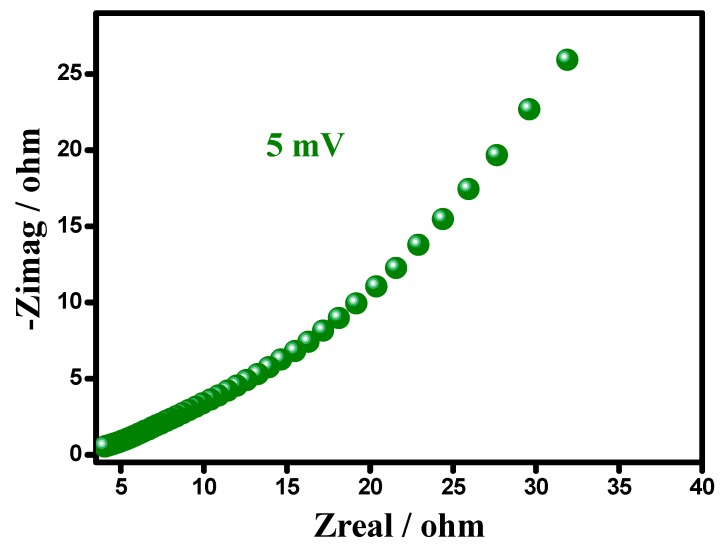
Nyquist plot of symmetric supercapacitor device.

Figure 13 shows the EIS result of the symmetric device, showing that the P–DBSA ║ P–DBSA supercapacitor exhibited a vertical line in the low frequency region, parallel to an imaginary axis, endorsing a highly capacitive character, and a fast double-layer formation, with a negligible Warburg resistance. Whereas the high frequency region resulted in an ESR and Rct. The device showed a low ESR value 3.01 Ω, attributed to the use of a wafer-thin separator, sandwiched between the two electrodes, providing a high permeability to ion. The separator resistance followed the equation: Rs = Ǫs.δs.S^−1^, where Ǫs is the permeability of the separator to ion, δs is the separator thickness and S is the surface area of the separator. Increase in the thickness of separator caused an increase in the ESR value. In addition, a low ESR value was very important to decrease the IR drop of the supercapacitors charged and discharged at high current densities. The diameter of the semicircle was the Rct at a high frequency region, indeed no semicircle was observed to have endorsed a fast electronic diffusion with a high conductivity.

**Figure 14:**
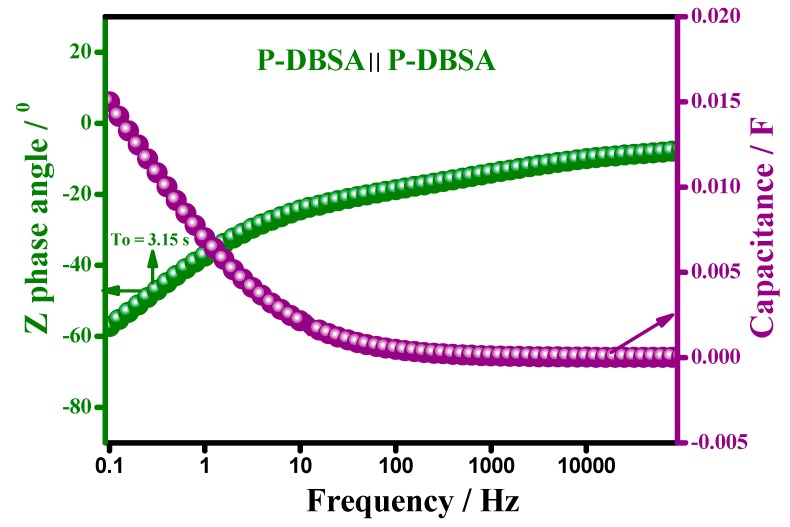
Variation of capacitance and the phase angle, with a change in frequency.

Figure 14 shows the Bode plot and capacitance compared to a frequency plot of a P–DBSA ║ P–DBSA supercapacitor device. The device was highly capacitive at a phase angle of −60°, resembling an ideal capacitive behavior, with a relaxation time constant (τo) of 3.15 s. Relaxation time constant (τo) was a significant factor for designing a supercapacitive device, its low value is desirable for a high capacitive performance, indicating a deeper penetration of ions in the highly porous morphology, as well a maximum capacitance that was achieved by a very fast charging. Capacitance versus frequency response was consistent with the τo value. The plot showed that a high capacitance of the device was retained in a broad frequency range, with more than 50% of the maximum capacitance being achieved, upto 10 Hz.

**Figure 15:**
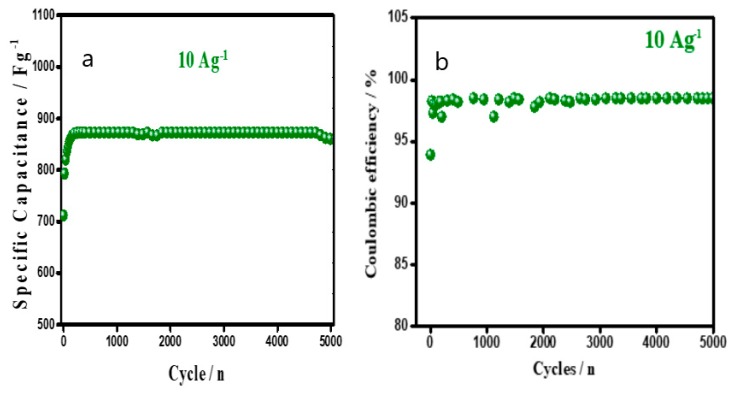
Variation of (**a**) specific capacitance and (**b**) coulombic efficiency of a P–DBSA║ P–DBSA device, with a continuous cycling life at 10 Ag^−1^.

The device was tested for stability by GCD, at a constant current density of 10 Ag^−1^ for 5000 cycles (Figure 15). For the first 200 cycles, the specific capacitance gradually increased by 25%, due to the activation of the inner material in the electrode. After 5000 consecutive cycles, the device retained an initial capacitance, indicating a longstanding stability and durability, as a stable electrode material for a supercapacitor. The columbic efficiency was retained till 98.5% after 5000 potential cycling (Figure 15b). The results suggested no counter ion drain effect and no degradation of electrode material, after cycling, and hence, endorsed a high conductivity of the electrode material with a longstanding stability.

### 3.6. Charge Storage Mechanism of a Supercapacitor Device

The charge storage/release during charging/discharging of a symmetrical device could be explained as follows. During the charging process, an increase in potential occur from V_o_ (initial potential = 0.0 V) to V_p_ (cut-off potential 0.75 V), by an injection of electrical charge on the 1st electrode (positive), which results in the transfer of the electrical charge, simultaneously, from the 2nd electrode (negative), inducing a reduction reaction accompanied by a de-doping process of the PANI. This causes a decrease in the potential of a negative electrode, from V_o_ (0.0 V) to Vn (−0.75 V), due to the release of a counter ion, into the electrolyte of the PANI chains. However, during the discharging process, an opposite process occurs, by releasing an electrical charge from a positive electrode, which is then returned to the initial potential value Vo. At the same time, an oxidation reaction is induced on a negative electrode, accompanied by a doping process of the PANI molecular chains. Consequently, the accumulation of anions on the negative electrode surface and protons, in the redox centers of the PANI chains, were collected, and the potential of the negative electrode increased again to reach an initial value Vo. Thus, the charge storage/ release, on the positive electrode, occurs only through surface accumulation, while the negative electrode is dominated by a redox reaction, as well as charge accumulation on the surface. On the above discussion, an overall cell reaction could be proposed as follows:
Positive:Pani + nA- + ne^+^ ‹—›Pani + (ne^+^) ⁞⁞ nA-[EDLC behavior]Negative:Pani_ox_ + ne- ‹—›Pani_red_ + nA- + nH^+^[PSC behavior]
where ‘A-‘ stands for the anion and ‘⁞⁞’ stands for the electric double layer, ‘e-‘ and ‘e+’ stand for the electron and positive elementary charge, respectively.

Therefore, the PANI of the positive electrode in a symmetric device, was mainly controlled by the EDLC mechanism (accumulation of charges on the surface) and did not play a role of a faradic source, although it was a redox material. While the charge storage/release process on the negative electrode was dominated by a redox reaction, and hence, followed the pseudocapacitive mechanism (Surface accumulated charge + bulk volume of PANI). Thus, the operation mechanism of the symmetric device constructed by P–DBSA ║ P–DBSA was asymmetric, which was an eminent feature of the deigning practical supercapacitor.

## 4. Conclusions

To encapsulate, the highly efficient PANI, doped with DBSA, was synthesized through a facile interfacial polymerization method. The material showed a high specific capacitance, excellent reversibility, a good rate capability, and a superior cycling stability, with a very low solution resistance (Rs = 0.61 Ω) and a potential drop (IR = 0.0191 V), as depicted from the CV, GCD, and EIS measurements. The binder free symmetrical device, fabricated by utilizing the synthesized PANI–DBSA as an electrode material, showed a prolonged calendar life, with a high energy density of 28 Whkg^−1^, at a power density of 285 Wkg^−1^ and was retained, as high as 15.1 Whkg^−1^, at a power density of 4500 Wkg^−1^. This successfully overwhelmed the tremendous challenge of low specific capacitance, low charge storage stability, fading of capacitance with current density growth, and need of a polymeric binder for pseudocapacitor electrode materials.

## Figures and Tables

**Table 1:** Comparison of specific capacitance values and percentage retention of polyaniline synthesized via different polymerization routes.

Synthesis Method	Electrolyte	Cs in Fg^−1^ (Current Density/Scan Rate)	% Retention (# Cycles)	Ref
Interfacial polymerization	1 M H_2_SO_4_	548 (0.18 Ag^−1^)	75 (1000)	[29]
Self-Assembly	1 M H_2_SO_4_	274 (1 Ag^−1^)	89 (1300)	[18]
Self-Assembly	1 M H_2_SO_4_	350 (1 Ag^−1^)	99 (500)	[18]
Soft template	1 M H_2_SO_4_	450 (0.5 Ag^−1^)	83 (10K)	
Self-Assembly	1 M H_2_SO_4_	455 (1 mVs^−1^)	65 (1300)	[32]
Self-Assembly	1 M H_2_SO_4_	625 (1 Ag^−1^)	77 (500)	[33]
Mechanochemical polymerization	1 M H_2_SO_4_	333 (1 Ag^−1^)	85 (1000)	[34]
Interfacial polymerization	1 M H_2_SO_4_	712 (0.5 Ag^−1^)	100 (5000)	**This Work**

## Supplementary Materials

The following are available online at https://www.mdpi.com/1996-1944/12/10/1626/s1, Figure S1: AFM image of PANI–DBSA., Table S1: FTIR band position of PANI–DBSA along with their assignments.
