# Supplementary material for: Highly Stable and Efficient Performance of Binder-Free Symmetric Supercapacitor Fabricated with Electroactive Polymer Synthesized via Interfacial Polymerization
Source: Materials (Basel). 2019 May 17;12(10):1626. doi: 10.3390/ma12101626 (PMC6567315; doi:10.3390/ma12101626)
Supplement: Supplementary file 1 [file materials-12-01626-s001.pdf]

materials

Supporting Information

Highly Stable and Efficient Performance of Binder-Free Symmetric Supercapacitor Fabricated with Electroactive Polymer Synthesized via Interfacial Polymerization

Muhammad Fahim [1], Anwar ul Haq Ali Shah [2] and Salma Bilal [1,3,*]

[1] National Centre of Excellence in Physical Chemistry, University of Peshawar,25120 Peshawar, Pakistan fahim_kn@yahoo.com

[2] Institute of Chemical Sciences, University of Peshawar, 25120 Peshawar, Pakistan anwarulhaqalishah@uop.edu.pk

[3] TU Braunschweig Institute of Energy and Process Systems Engineering, Franz-Liszt-Straße 35, 38106 Braunschweig, Germany

* Correspondence: s.bilal@tu-braunschweig.de or dresalmabilal@gmail.com; Tel.: +0049-531-39163651 or +0092-919216766

Received: 22 April 2019; Accepted: 15 May 2019; Published: date

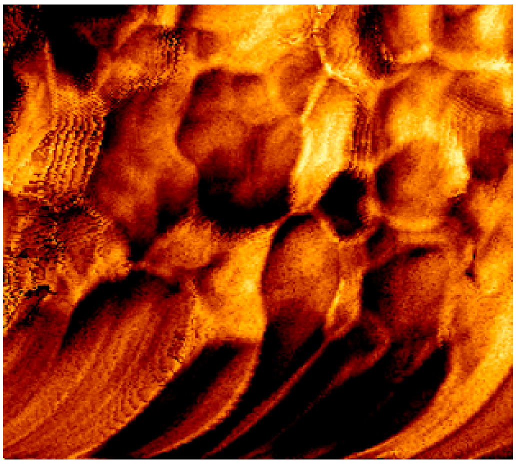

Figure S1. AFM image of PANI–DBSA.

Table S1. FTIR band position of PANI–DBSA along with their assignments.

Assignment	Band position (cm^{-1})
Secondary bounded NH stretch of aromatic amine	3213
Aromatic C—H stretch	2931
Alkene C—H stretch	2907
Aliphatic C—H stretch	2835
Quinoid ring (Q)	1552
Benzenoid ring (B)	1479
C-N stretch in QBQ, QBB, BBQ	1282
Electronic like absorption of N=Q=N	1146
SO_3 stretch	
Symmetric SO_2 stretch	1124
P=O stretch	
S=O stretch	1024
Aromatic C-H out of plan bending (para substituted benzene)	789
C—S stretch	

S—O stretch	665
N—H out of plane bending	503
(IQ/IB)	1.05

References

1. Wang, X.; Liu, D.; Deng, J.; Duan, X.; Guo, J.; Liu, P. Improving cyclic stability of polyaniline by thermal crosslinking as electrode material for supercapacitors. RSC Adv. 2015, 5, 78545–78552.
2. Gawli, Y.; Banerjee, A.; Dhakras, D.; Deo, M.; Bulani, D.; Wadgaonkar, P.; Shelke, M.; Ogale, S. 3D Polyaniline Architecture by Concurrent Inorganic and Organic Acid Doping for Superior and Robust High Rate Supercapacitor Performance. Sci. Rep. 2016, 6, 21002.
3. Huang, J.; Virji, S.; Weiller, B.H.; Kaner, R.B. Polyaniline Nanofibers: Facile Synthesis and Chemical Sensors. J. Am. Chem. Soc. 2003, 125, 314–315.
4. Tissera, N.D.; Wijesena, R.N.; Rathnayake, S.; de Silva, R.M.; de Silva, K.M.N. Heterogeneous in Situ Polymerization of Polyaniline (PANI) Nanofibers on Cotton Textiles: Improved Electrical Conductivity, Electrical Switching, and Tuning Properties. Carbohydr. Polym. 2018, 186, 35–44.
5. Chen, J.; Chao, D.; Lu, X.; Zhang, W. Novel Interfacial Polymerization for Radially Oriented Polyaniline Nanofibers. Mater. Lett. 2007, 61, 1419–1423.
6. Ding , S; Mao, H; Zhang, W. Fabrication of DBSA-doped polyaniline nanorods by interfacial polymerization. J. Appl. Polym. Sci. 2008, 109, 2842–2847.
7. Oueiny, C.; Berlioz, S.; Perrin, F.X. Assembly of Polyaniline Nanotubes by Interfacial Polymerization for Corrosion Protection. Phys. Chem. Chem. Phys. 2016, 18, 3504–3509.
8. Ji, T.; Cao, W.; Chen, L.; Mu, L.; Wang, H.; Gong, X.; Lu, X.; Zhu, J. Confined Molecular Motion across Liquid/Liquid Interfaces in a Triphasic Reaction towards Free-Standing Conductive Polymer Tube Arrays. J. Mater. Chem. 2016, 4, 6290–6294.
9. Bilal, S.; Gul, S.; Holze, R.; Shah, A.A. An impressive emulsion polymerization route for the synthesis of highly soluble and conducting polyaniline salts. Synth. Met. 2015, 206, 131–144.

© 2019 by the authors. Submitted for possible open access publication under the terms and conditions of the Creative Commons Attribution (CC BY) license (http://creativecommons.org/licenses/by/4.0/).
